# Evaluation of Strength and Irradiated Movement Pattern Resulting from Trunk Motions of the Proprioceptive Neuromuscular Facilitation

**DOI:** 10.1155/2012/281937

**Published:** 2012-10-04

**Authors:** Luciana Bahia Gontijo, Polianna Delfino Pereira, Camila Danielle Cunha Neves, Ana Paula Santos, Dionis de Castro Dutra Machado, Victor Hugo do Vale Bastos

**Affiliations:** ^1^Physical Therapy Department, Federal University of the Valleys of Jequitinhonha and Mucuri, 39100-000 Diamantina, MG, Brazil; ^2^Department of Neuroscience and Behavioral Sciences, School of Medicine of Ribeirão Preto, University of São Paulo, 14049-900 Ribeirão Preto, SP, Brazil; ^3^Multicenter Post Graduation Program in Physiological Sciences, Federal University of the Valleys of Jequitinhonha and Mucuri, 39100-000 Diamantina, MG, Brazil; ^4^Physical Therapy Department, Brain Mapping Lab & Functionality, Federal University of Piauí, 64202-020 Parnaíba, PI, Brazil; ^5^CNPq, 71605-001 Brasília, DF, Brazil

## Abstract

*Introduction*. The proprioceptive neuromuscular facilitation (PNF) is a physiotherapeutic concept based on muscle and joint proprioceptive stimulation. Among its principles, the irradiation is the reaction of the distinct regional muscle contractions to the position of the application of the motions. *Objective*. To investigate the presence of irradiated dorsiflexion and plantar flexion and the existing strength generated by them during application of PNF trunk motions. *Methods*. The study was conducted with 30 sedentary and female volunteers, the PNF motions of trunk flexion, and extension with the foot (right and left) positioned in a developed equipment coupled to the load cell, which measured the strength irradiated in Newton. *Results*. Most of the volunteers irradiated dorsal flexion in the performance of the flexion and plantar flexion during the extension motion, both presenting an average force of 8.942 N and 10.193 N, respectively. *Conclusion*. The distal irradiation in lower limbs became evident, reinforcing the therapeutic actions to the PNF indirect muscular activation.

## 1. Introduction

Proprioceptive neuromuscular facilitation (PNF) is a concept of treatment [1] in which the basic philosophy considers that every human, including those with disabilities, has an untapped existing potential [2]. PNF is a method used in clinical practice [3] in order to improve development of neuromuscular system by stimulation of muscle and joint proprioceptors [4]. Some concepts characterize the philosophy under the technique: integrated approach (i.e., treatment is directed toward the human as a whole and not only as a body segment), based on an untapped existing potential (mobilizing reserves patients), positive approach (reinforcing patient's ability on a physical and psychological level) whose goal is reaching the level of function from this patient through the International Classification of Functioning (ICF) model. 

Among the PNF's principles, irradiation is a useful aspect for patients with muscle weakness in areas that cannot be directly worked (strengthened) [5]. This principle is based on fact that stimulation of strong and preserved muscle groups produces strong activation of injured and weak muscles, facilitating muscle contraction [6]. So, these weak muscles can develop an increase in the duration and/or intensity by the spread of the response to stimulation or by the synergistic muscle inhibition [7]. Some studies have investigated the presence of irradiation [3, 7–9], but type of muscle (agonist or antagonist) which receives irradiation is not consistent in the literature. According to Sherrigton [7], irradiation only innervates agonist muscles, but Hellebrandt et al. [8] found in their studies that during wrist exercises, the most significant effects were seen in contralateral limb muscles [9]. In order to determine quantitatively and accurately the existence of muscle strength, accurate equipment must be used.

To evaluate force exerted on limb by virtue of irradiation, it is possible to use load cell equipment as force measurement transducer due to its lower cost and greater portability in addition to being one of the most used instruments for measuring force [10–14]. The operating principle of load cells is based on variations in the ohmic resistance of a sensor named as extensometer or strain gauge, when submitted to a deformation. Specialized tools for verifying irradiation to the lower limbs have not been found on the market and in the literature; therefore a specific apparatus was developed for this purpose. 

PNF is one of the main concepts of rehabilitation treatment for patients with neurological injuries, being used for several years and spread by known authors such as Kabat, Susan, who defend its efficiency. The trunk is the central region for motor control of lower and upper limbs and can irradiate to them. When an injury of nervous system occurs, as a stroke, this motor control can be disturbed and does not allow effective movements at limbs [1, 2]. However, researches are still scarce nowadays, especially regarding the neurophysiological basis of the irradiation principle. Further studies are necessary to form a more concrete and detailed definition, which can trigger improvements in physical conditions and life quality for the patient, avoiding its erroneous applications. In this sense, the study objective was to evaluate strength, using load cell, and motion pattern (dorsiflexion or plantar flexion) triggered by irradiation resulting from PNF motions of trunk flexion and extension.

## 2. Methodology

### 2.1. Subjects

Sample of this cross-sectional study consisted of 30 female volunteers aged 18–30, university students, sedentary, who performed less than 20 minutes of physical activity in less than 3 days in a week, in the last six months [15]. The selection for only female volunteers occurred due to the fact that the researcher was a woman, which would prevent performance of the maximum strengthening from male volunteers since they possibly have higher force range. Smokers, male gender, subjects with presence of any cardiac, pulmonary, musculoskeletal, or neurological diseases, with functional limitation to perform resistance exercises or those having baseline blood pressure above 140/90 mmHg, were excluded. This study covers standards required for researches involving human subjects contained in Declaration of Helsinki and was approved by the Ethics Committee in Research of UFVJM under protocol 132/10, also in accordance with the Resolution CNS 196/96.

All volunteers were evaluated about body mass index according to the criteria of the National Health and Nutrition Examination Survey, proposed in United States and to blood pressure, using the same aneroid and stethoscope sphygmomanometer type, and answered the item related to the lower limb of the Oldfield Handedness Inventory [16]. Subjects were not familiar with the PNF concept to prevent possible bias. Volunteers were instructed not to drink alcohol 24 hours before evaluation, not to ingest caffeine (chocolate, chocolate drinks, coffees, teas, soft drinks, and the like) on the day of evaluation, to have a good sleep on previous night, and not practice any physical activity 24 hours before evaluation. All subjects eligible participated in the study; no desistance occurred.

### 2.2. Measurement

**Figure 1:**
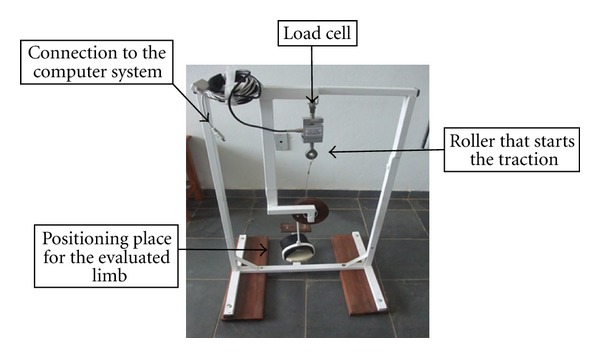
Measuring instrument, coupled to the load cell, used to measure the irradiation and the force generated by the PNF motions.

**Figure 2:**
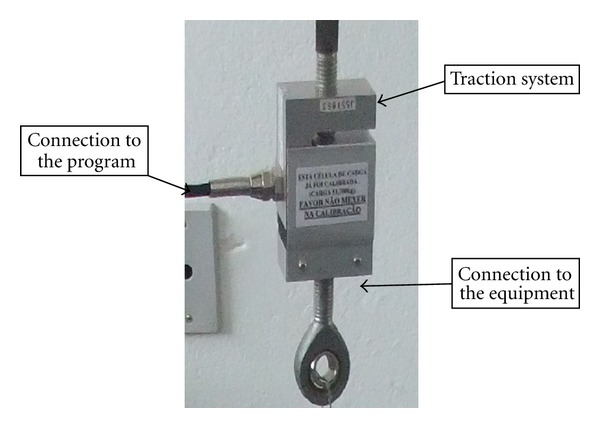
Load cell.

The instrument used to measure plantar flexion and dorsiflexion pattern (Figure 1) and the force generated by irradiation was developed by researchers with the help of a designers and its efficiency was tested. The device enables foot positioning and starts the rolling to right (indicating dorsiflexion) or to left (indicating plantar flexion) pulling the load cell coupled to the system, generating its deformation and the measurement in a computerized system. The load cell used was from Miotec-Biomedical Equipment 250 kg (Figure 2).

**Figure 3:**
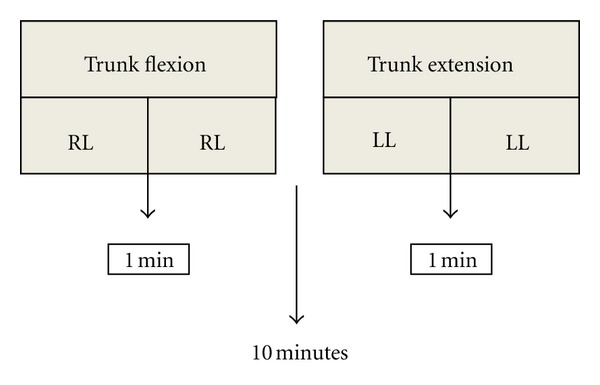
Performed motion sequence. RL: right limb; LL: left limb.

 The data was collected in one single moment, according to volunteers availability, at morning or at afternoon. A familiarity with the motions was performed 48 hours before the study, but it did not involve any learning due to the given interval. Each volunteer seated at the same wooden chair with a backrest and without armrests, with the foot on the system, the knee flexion at 60° degrees on a foam wedge. Trunk flexion and extension motions were performed using the resistance PNF motion, evaluating both positioning feet, the left and the right one on the system at each motion. The motion of each foot was held in two trials, with one minute rest between them, considering the highest value and standard developed by this value. Between trunk flexion and extension motions, a ten-minute break was given in order to avoid fatigue and the decrease of the maximum developed force (Figure 3).

### 2.3. Motions

The task was explained to each subject and started with the examiner's command “go.” This command is an auditory stimulus and works like a positive reinforcement. The command was repeated some times during the task execution according basic to principles of PNF's concept (positive approach). The motions followed the concepts established by PNF's philosophy [1], in which a continuous manual resistance was applied through lumbrical contact, and an encouragement of voice command was given. Then, the volunteers performed motions of trunk flexion and extension, using their maximum strength. The quantification of maximum strength developed by volunteers was made by overcoming theraband elastic resistance, in which the intensity of force was represented by the following: entire elastic in purple, in gray, and in orange, folded elastic in purple, in gray, and in orange. 

**Figure 4:**
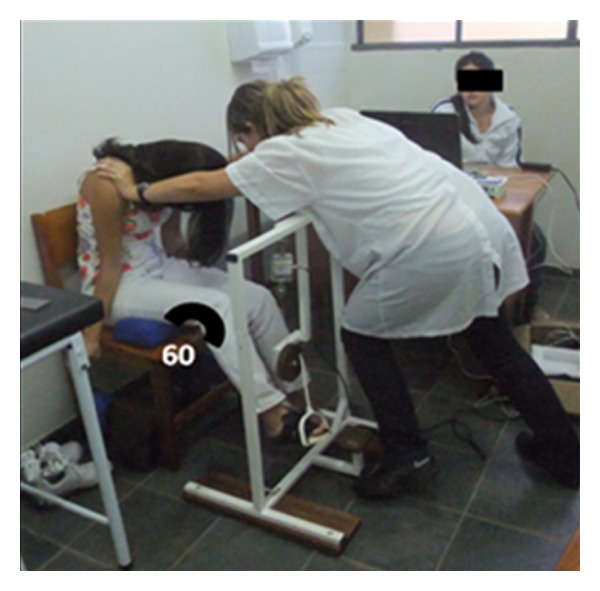
Trunk flexion motion.

Trunk flexion motion was performed with therapist in front of the volunteer, with the elbows extended and one leg in front of the other, with the knees in semiflexion, resting both hands on anterosuperior surface of the volunteer trunk, with lumbrical grip at anterior shoulder region. The volunteer was sitting on a wooden chair, and the limb was evaluated positioned on equipment with knee flexion of 60°. The movement started from neutral position of trunk (upright position) followed by trunk flexion until it reaches 45° against a manual resistance from the therapist (Figure 4).

**Figure 5:**
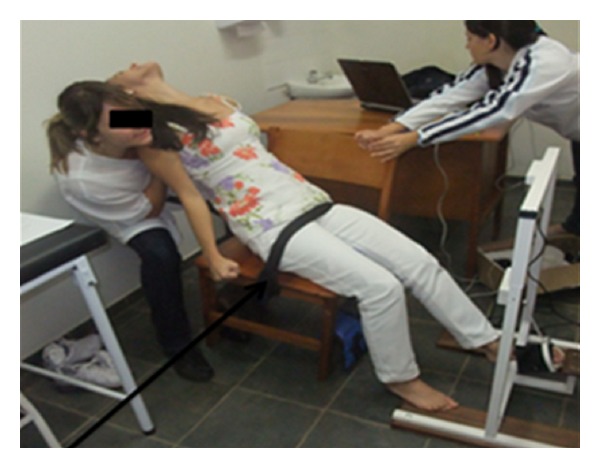
Trunk extension motion.

Extension motion was performed with therapist behind the volunteer, extended elbows and one leg in front of the other, with the knees in semiflexion, resting with lumbrical grip at posterior shoulder region. The volunteer was sitting on a chair and involved with a Velcro strip to prevent sliding on the seat during the motion. The movement started from neutral position of trunk and performed an extension until it reaches 45° against the manual resistance from the therapist, so that the volunteer could perform her maximum strength. The remaining positions were the same as the flexion motion (Figure 5).

To perform statistical analysis the Statistical Package for the Social Sciences (SPSS) was used. The Shapiro-Wilk test verified the nonnormality from the data of the strength generated in each shift (morning and afternoon) and limb (right and left), so the comparison of the averages was performed using nonparametric Mann-Whitney test. Normality was found for data of motion (extension and flexion), so the independent *t*-test was performed to compare the averages. Values less than 0.05 were considered as statistically significant, according to the Fisher criteria.

## 3. Results

 The sample was composed of 30 subjects with mean age of 22.57 years old, weight of 57.42 kg, height of 164.23 cm, and BMI equivalent to 21.30 (Table 1). 

The execution of trunk flexion motion caused irradiation to dorsal flexion at 96.7% of subjects when right foot was placed on equipment and at 100% when the left foot was positionated. During extension motion, plantar flexion irradiation was generated at 100% of subjects in both of the positioned feet. The dorsiflexion developed an average force of 2.60N on the right limb and 2.84N on the left limb, and the triggered plantar flexion generated an average force of 7.52N on the right limb and of 7.24N on the left one. 

From the total sample, 14 volunteers participated at morning and 16 at afternoon.  Table 2 shows the mean strength that was triggered by each motion pattern (flexion or extension trunk) with both feet positioning. Comparing this data, higher mean strength generated by volunteers that performed the motion of the right limb at morning was considered significant. It presented *P* = 0.013 in the plantar flexion of the right foot triggered by trunk extension, and *P* = 0.029 on the right foot dorsiflexion triggered by trunk flexion.

Stronger subjects used therabands of higher resistance and so, more force was irradiated. However, the results from these force descriptions will not be presented in this study due to the large number of variables already available, which need the development of new future researches. The laterality questionnaire obtained dominance of the right lower limb in 100% of the volunteers.

The independent *t*-test was used to compare the total force developed by the flexion and extension motions regardless of the positioned foot. At trunk flexion mean was 2.72N (±2.40), and at trunk extension it was 7.38N (±5.61). Significant values were found in relation to a greater force generated by the extension (*P* = 0.000).

## 4. Discussion

 This study aimed to investigate the pattern (dorsiflexion or plantar flexion) and the amount of irradiated strength resulting from the resistance imposed on trunk flexion and extension movement of PNF. The sample was composed of female subjects in which smallest amount of lean mass observed [17] may have reduced the average of the irradiated force. Further studies with male volunteers should be conducted to investigate this hypothesis. This study presents initial data as the first surveys from future researches that are intended to be carried out by the researchers. 

 Trunk flexion motion significantly irradiated dorsal flexion movement for both right and left ankle. Possibly a need of approximation from the origin and the insertion of the rectus femoris favored the great length tension required in sufficient pelvic stabilization for trunk flexion in its maximum efficiency [18], considering, during the data collection, an associated ipsilateral hip flexion with dorsiflexion was observed. So, the contraction of hip flexors could lead to shortening of deep lateral myofascial causing ankle dorsiflexion [19]. This fact resembles the primitive patterns of sensory inputs during walking in basic patterns, specifically the swing phase [20]. Therefore, the irradiated movement may be associated to the triggering stimulus of nervous system, which presents among its circuits predetermined sequential activation of muscle contractions to the achievement of efficient walking and lower energy expenditure [21]. 

It is noteworthy that the present study used a simple technique of trunk flexion and extension to observe irradiation to the lower limbs; another possibility consists in using trunk rotation to cause irradiation to them. In physical therapy practice, irradiation can be used in cases where paresis is noted; that is, stroke or spinal cord injury and other conditions present with weakness by immobility. However, integrity of trunk movements is necessary. If trunk does not have sufficient strength or have an instability, it must be worked on previously to acquire strength and stability sufficient to generate irradiation to the lower limbs.

 During trunk extension motion, the movement performed by the ankle was the plantar flexion, on both limbs that were analyzed. A similar pattern triggered by the primitive patterns of walking (pulse phase) was present [21], but initial position of the volunteer (hip and knee flexion) led to changes in patterns considered normal. This event is held by the human body as adapters to atypical stimulus [22, 23]. In this way, trunk extension performed by the erector spinae triggered a hip extension and plantar flexion. However, instead of having knee flexion there was a trend to extension, favoring the biomechanical advantage of rectus femoris contraction as soon as it approximated its insertions, so that it could eccentrically stabilize the pelvis [18]. Since the developed standard was significant in the study, the trunk motions may be directed to the “indirect” strengthening, in which it starts the trunk flexion training to strengthen the dorsal flexors and the plantar flexors with trunk extension. 

The origination of higher force ranges in the right limb to perform the task may be related to high incidence of volunteers right-handedness, observed by Oldfield inventory. Studies believe that dominant lower limb has a higher efficiency of force generation [24, 25] since the asymmetric functions are specified in the mobility to perform unipodal tasks due to more complex neuromuscular demands [26]. This event has been explained by neurodevelopmental theory which believes that the influence of asymmetry of ear and labyrinth development, in third trimester of pregnancy, is related to higher efficiency of ipsilateral hemisphere for providing early experience [27]. Another aspect is the fact that social culture tends to create demand for tasks with right limb, favoring its postural control and, consequently, a more efficient contraction [26]. Strength generation itself, as demonstrated numerically in joules, proves the existence of distal strength in irradiation. Therefore, the studied motions can be applied to perform muscle contraction without direct contact to the activated muscle.

One of the hypotheses to explain better results at morning is that the circadian rhythm might have been influenced because the best development of skeletal muscles occurs as a metabolic consequence of human, which in most cases are more activated by specific hypothalamic hormones [28]. The hormonal peaks vary during the 24 hours of the day for mammals, according to the energy required from the species [29], increasing metabolism in skeletal muscle, which can help effectiveness of its contraction. According to Atkinson [30] this condition is more constant at morning, which may have influenced higher levels of strength during this period, as well as it is possible that the volunteers who performed the motions at afternoon had done more activities during the day, compared to the ones from the morning shift, leading to a greater cumulative muscle fatigue. In accordance with these results, training aiming to achieve higher thresholds of strength will have a greater efficiency at morning. In this study the maneuvers application at afternoon was necessary due to samples availability and was not a study limitation. Another hypothesis that can be explored is examiner's fatigue at afternoon, but on research design was programmed the routine data collection considering examiner rested to apply the correct technique, and it was followed.

The plantar flexion produced a higher average of force range as a consequent movement from trunk extension motion. Newton's Law of Universal Gravitation may have influenced the result of the unleashed force [31, 32], since the volunteer had the sum of the gravity and muscle strengths in the plantar flexion of the determined position. Different from dorsiflexion, that had the force of gravity as a negative element. Another determining factor may have been the combination of gravity to muscle aspect with higher numbers of muscle fibers and muscle favorable biomechanics (by semiflexion of the knee from the initial position of the test), leading to an intrinsic advantage of the physiological mechanisms of mentioned muscle [18, 33]. In order to develop the equipment, some items were established as essential in its design. The possibility of dorsal or plantar flexion execution was one of these items, since it was necessary to have them primarily investigated because of its nondetermination in the literature. However, its application had the influence of gravitational force as a consequence, which can be disregarded by mathematical calculations in future analyses.

## 5. Conclusion

The results show that trunk flexion and extension motions generate an irradiated movement in dorsiflexion and plantar flexion, respectively. Considerable strength values were measured as a result of the indirect muscle activation of the muscle groups responsible for such action, both in healthy and sedentary women. Therefore, PNF concept enables the performance of muscle activation of dorsal and plantar flexors which cannot be worked directly as in poststroke hemiparetic members.

## Figures and Tables

**Table 1:** Sample description.

	Age	Weight	Height	BMI
Average	22.57	57.42	164.23	21.30
Standard deviation	1.695	7.238	5.52	2.38
Maximum	27	73	178.00	27.62
Minimum	20	43	154.00	17.33

**Table 2:** Strength generated in each shift.

Motion pattern	Shift	Mean strength	Standard deviation
Flexion—right foot	Morning	3.67	0.77
Flexion—right foot	Afternoon	1.67	0.36
Flexion—left foot	Morning	3.77	0.74
Flexion—left foot	Afternoon	2.03	0.44
Extension—right foot	Morning	9.71	1.48
Extension—right foot	Afternoon	5.60	1.41
Extension—left foot	Morning	9.02	1.44
Extension—left foot	Afternoon	5.69	1.26

## References

[1] Susan S, Beckers AD, Buck M, Heidelberg MV (2003). *FNP in Practice: An Illustrated Guide*.

[2] Youdas JW, Arend DB, Exstrom JM, Helmus TJ, Rozeboom JD, Hollman JH (2012). Comparison of muscle activation levels during arm abduction in the plane of the scapula vs.proprioceptive neuromuscular facilitation upper extremity patterns. *The Journal of Strength & Conditioning Research*.

[3] Meningroni PC, Nakada CS, Hata L, Fuzaro AC, Júnior WM, Araujo JE (2009). Contralateral force irradiation for the activation of tibialis anterior muscle in carriers of Charcot-Marie-Tooth disease: effect of PNF intervention program. *Revista Brasileira de Fisioterapia*.

[4] Sharma KN (2012). *Handbook of Proprioceptive Neuromuscular Facilitation: Basic Concepts and Techniques*.

[5] Pink PMS (1981). Contralateral effects of upper extremity proprioceptive neuromuscular facilitation patterns. *Physical Therapy*.

[6] Kofotolis N, Vrabas IS, Vamvakoudis E, Papanikolaou A, Mandroukas K (2005). Proprioceptive neuromuscular facilitation training induced alterations in muscle fibre type and cross sectional area. *British journal of sports medicine*.

[7] Sherrington C (1947). *The Integrative Action of the Nervous System*.

[8] Hellebrandt FA, Houtz SJ, Partridge MJ, Walters CE (1956). Tonic neck reflexes in exercise of stress in man. *American Journal of Physical Medicine and Rehabilitation*.

[9] Hellebrandt FA, Waterland JC (1962). Indirect learning. The influence of unimanual exercise on related muscle groups of the same and the opposite side. *American Journal of Physical Medicine*.

[10] Loss J (1998). Recommended method for correlating muscle strength and electromyography. *Movement*.

[11] Schettino L (2007). Comparative study on the strength and autonomy of sedentary versus active elderly women. *Revista Terapia Manual*.

[12] Papoti M (2003). Standardization of a specific protocol for determining the anaerobic conditioning in swimmers using load cells. *Revista Portuguesa de Ciências do Desporto*.

[13] Gonçalves M, Barbosa FSS (2005). Analysis of strength and resistance parameters of the lumbar spinae erector muscles during isometric exercise at different effort levels. *Revista Brasileira de Medicina do Esporte*.

[14] Miller JM, Miller JAA, Perruchini D, DeLancey JOL (2007). Test-retest reliability of an instrumented speculum for measuring vaginal closure force. *Neurourology and Urodynamics*.

[15] Jakicic JM, Marcus BH, Gallagher KI, Napolitano M, Lang W (2003). Effect of exercise duration and intensity on weight loss in overweight, sedentary women: a randomized trial. *Journal of the American Medical Association*.

[16] Elias LJ, Bryden MP, Bulman-Fleming MB (1998). Footedness is a better predictor than is handedness of emotional lateralization. *Neuropsychologia*.

[17] Okamoto Y, Kunimatsu A, Kono T, Kujiraoka Y, Sonobe J, Minami M (2010). Gender differences in MR muscle tractography. *Magnetic Resonance in Medical Sciences*.

[18] Neumann AD (2011). *Kinesiology of the Musculoskeletal System*.

[19] Myers T (2010). *Anatomy Trains*.

[20] Frigon A, Gossard JP (2010). Evidence for specialized rhythm-generating mechanisms in the adult mammalian spinal cord. *Journal of Neuroscience*.

[21] Ivanenko YP, Poppele RE, Lacquaniti F (2004). Five basic muscle activation patterns account for muscle activity during human locomotion. *Journal of Physiology*.

[22] Cappellini G, Ivanenko YP, Dominici N, Poppele RE, Lacquaniti F (2010). Motor patterns during walking on a slippery walkway. *Journal of Neurophysiology*.

[23] Cham R, Redfern MS (2002). Changes in gait when anticipating slippery floors. *Gait and Posture*.

[24] Elias LJ, Bryden MP, Bulman-Fleming MB (1998). Footedness is a better predictor than is handedness of emotional lateralization. *Neuropsychologia*.

[25] McLean BD, Tumilty MD (1993). Left-right asymmetry in two types of soccer kick. *British Journal of Sports Medicine*.

[26] Gabbard C, Hart S (1996). A Question of foot dominance. *Journal of General Psychology*.

[27] Previc FH (1991). A general theory concerning the prenatal origins of cerebral lateralization in humans. *Psychological Review*.

[28] Zhang X, Dube TJ, Esser KA (2009). Working around the clock: circadian rhythms and skeletal muscle. *Journal of Applied Physiology*.

[29] Almon RR, Yang E, Lai W (2008). Relationships between circadian rhythms and modulation of gene expression by glucocorticoids in skeletal muscle. *American Journal of Physiology—Regulatory Integrative and Comparative Physiology*.

[30] Atkinson G, Jones H, Ainslie PN (2010). Circadian variation in the circulatory responses to exercise: relevance to the morning peaks in strokes and cardiac events. *European Journal of Applied Physiology*.

[31] Zatsiorsky VM, Gao F, Latash ML (2005). Motor control goes beyond physics: differential effects of gravity and inertia on finger forces during manipulation of hand-held objects. *Experimental Brain Research*.

[32] Baltzopoulos V, Brodie DA (1989). Isokinetic dynamometry. Applications and limitations. *Sports Medicine*.

[33] Degens H, Erskine RM, Morse CI (2009). Disproportionate changes in skeletal muscle strength and size with resistance training and ageing. *Journal of Musculoskeletal Neuronal Interactions*.

